# Endoscopic ultrasound-guided hepaticogastrostomy with a 22-G needle and novel double-lumen catheter after pancreaticoduodenectomy

**DOI:** 10.1093/gastro/goag097

**Published:** 2026-09-18

**Authors:** Toru Kaneko, Mitsuhiro Kida, Takahiro Kurosu, Yuta Sakabe, Shiori Koyama, Hisashi Hidaka, Chika Kusano

**Affiliations:** Department of Gastroenterology, Kitasato University Medical Center, 6-100 Arai, Kitamoto, Saitama, 364-8501, Japan; Department of Gastroenterology, Kitasato University Hospital, 1-1-15 Kitasato, Minami Sagamihara, Kanagawa, 252-0375, Japan; Department of Gastroenterology, Kitasato University Medical Center, 6-100 Arai, Kitamoto, Saitama, 364-8501, Japan; Department of Gastroenterology, Kitasato University Hospital, 1-1-15 Kitasato, Minami Sagamihara, Kanagawa, 252-0375, Japan; Department of Gastroenterology, Kitasato University Medical Center, 6-100 Arai, Kitamoto, Saitama, 364-8501, Japan; Department of Gastroenterology, Kitasato University Hospital, 1-1-15 Kitasato, Minami Sagamihara, Kanagawa, 252-0375, Japan; Department of Gastroenterology, Kitasato University Medical Center, 6-100 Arai, Kitamoto, Saitama, 364-8501, Japan; Department of Gastroenterology, Kitasato University Hospital, 1-1-15 Kitasato, Minami Sagamihara, Kanagawa, 252-0375, Japan; Department of Gastroenterology, Kitasato University Medical Center, 6-100 Arai, Kitamoto, Saitama, 364-8501, Japan; Department of Gastroenterology, Kitasato University Hospital, 1-1-15 Kitasato, Minami Sagamihara, Kanagawa, 252-0375, Japan; Department of Gastroenterology, Kitasato University Medical Center, 6-100 Arai, Kitamoto, Saitama, 364-8501, Japan; Department of Gastroenterology, Kitasato University Hospital, 1-1-15 Kitasato, Minami Sagamihara, Kanagawa, 252-0375, Japan; Department of Gastroenterology, Kitasato University Hospital, 1-1-15 Kitasato, Minami Sagamihara, Kanagawa, 252-0375, Japan

## Introduction

Endoscopic ultrasound (EUS)-guided hepaticogastrostomy (HGS) is an established alternative when endoscopic retrograde cholangiopancreatography (ERCP) is not feasible, particularly in patients with surgically altered anatomy [1, 2]. However, puncture using a 19-G needle can be technically challenging in cases with a small intrahepatic bile duct or when the maneuverability of the EUS endoscope is limited, including situations in which adequate scope angulation or effective use of the elevator cannot be achieved. EUS-HGS using a 22-G needle has been reported as an effective alternative approach in patients with a small or angulated intrahepatic bile duct and in situations where scope maneuverability is limited [3]. Compared with a 19-G needle, the 22-G needle provides a smaller puncture profile and greater flexibility of the needle axis, which may facilitate puncture and scope positioning. However, the 0.018-inch guidewire (GW) may lack sufficient stiffness for tract dilation and stent deployment. Compared with a conventional 0.025-inch GW, the smaller 0.018-inch GW provides less support during device exchange, which may compromise procedural stability and make subsequent tract dilation and stent deployment more technically demanding. The double-guidewire technique (DGT), in which an additional 0.025-inch GW is placed via a double-lumen catheter (DLC), has been introduced to improve GW support, axis stability, and device delivery [4]. However, conventional DLCs frequently require prior tract dilation, potentially increasing the risk of bile leakage. A recently developed novel DLC (Supplementary Fig. S1; Uneven double-lumen catheter 18 type; PIOLAX Medical, Kanagawa, Japan) is compatible with a 0.018-inch GW and features a tapered tip with a minimal step-off, a 6-Fr shaft, and an additional lumen located 5 mm proximal to the tip that permits insertion of a second GW. Unlike conventional dilators, this device functions as a flexible contrast catheter that allows GW manipulation and contrast injection while facilitating rapid establishment of the DGT without prior tract dilation, which may facilitate fine-gauge EUS-HGS in selected cases. Herein, we report a case of EUS-HGS performed using a 22-G needle combined with DGT facilitated by this novel uneven DLC (Supplementary Video S1).

## Case presentation

**Figure 1:**
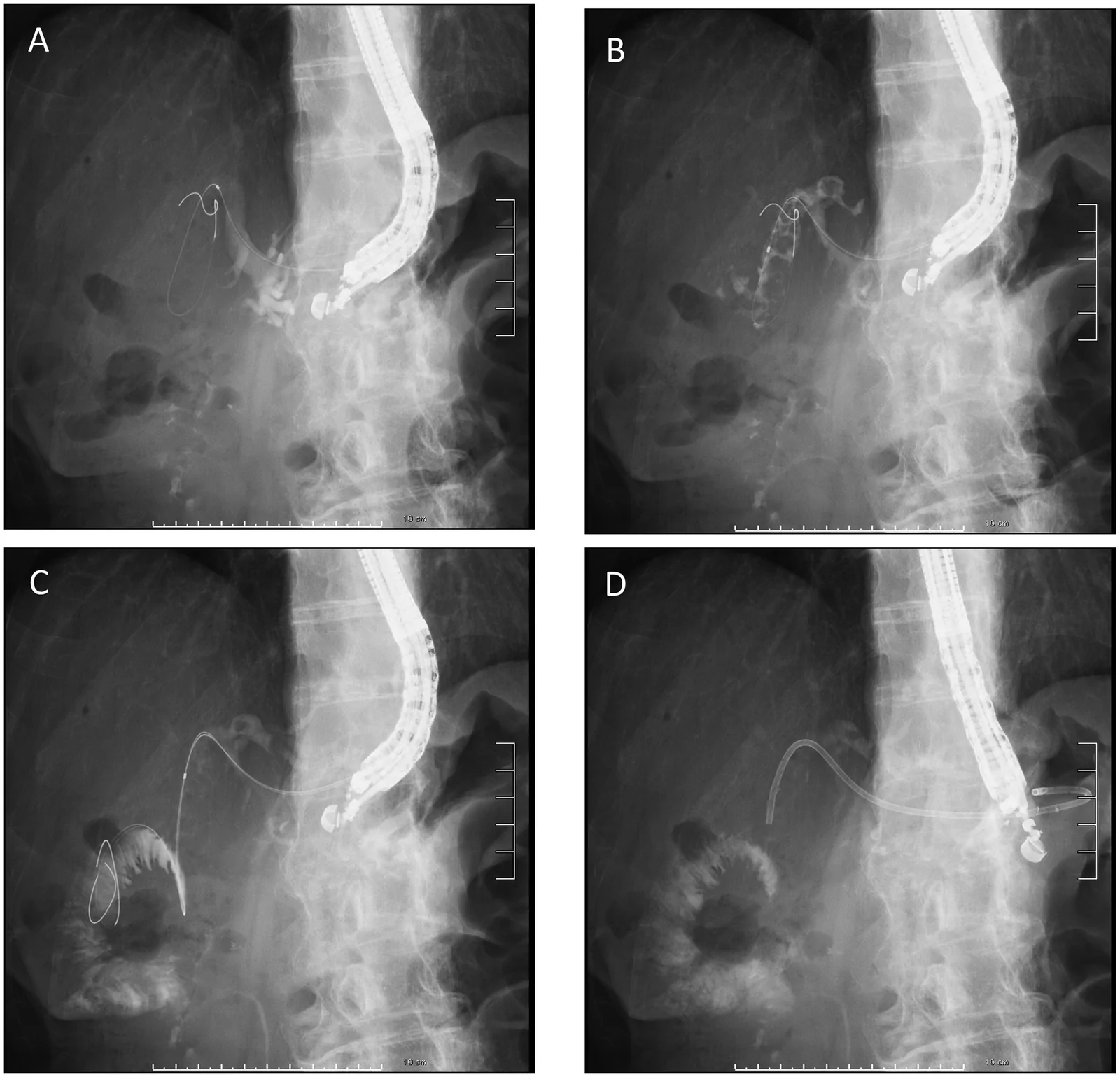
Endoscopic ultrasound (EUS)-guided hepaticogastrostomy using a 22-G needle and a novel double-lumen catheter. (A) Puncture of the B3 intrahepatic bile duct using a 22-G needle under EUS guidance, followed by contrast medium injection and placement of a 0.018-inch guidewire in the bile duct. (B) Advancement of the novel double-lumen catheter over a 0.018-inch guidewire without prior tract dilation. (C) Placement of an additional 0.025-inch guidewire through the novel double-lumen catheter using the double-guidewire technique. (D) Placement of a 7-Fr plastic stent in the bile duct without additional dilation.

A 75-year-old man with a history of pancreaticoduodenectomy presented with acute cholangitis caused by intrahepatic bile duct stones (Supplementary Fig. S2A and B). Balloon-assisted ERCP failed owing to the inability to reach the anastomotic site. Consequently, EUS-HGS was performed. The B3 duct was selected because it provided a stable transgastric puncture route, a favorable puncture angle, and no intervening vessels, whereas B2 was avoided to minimize the risk of transesophageal puncture. The diameter of the punctured B3 duct was ∼5 mm. Under EUS guidance, the B3 intrahepatic bile duct was punctured using a 22-G needle (Fig. 1A), and a 0.018-inch GW was placed. The novel DLC was advanced without prior tract dilation (Fig. 1B). A second 0.025-inch GW was inserted to establish the DGT (Fig. 1C). Subsequently, a 7-Fr, 14-cm single-pigtail plastic stent (Type IT stent; Gadelius Medical, Tokyo, Japan) was successfully deployed without additional dilation (Fig. 1D, Supplementary Video S1). The total procedure time was 16 min. No adverse events occurred, and the patient’s cholangitis resolved promptly.

## Discussion

A recent report has described fine-gauge EUS-HGS using a double-lumen dilator [5]. Such dilator-based devices are primarily designed for tract dilation and, therefore, require sufficient stiffness. In contrast, the novel DLC functions as a flexible contrast catheter, which may facilitate catheter advancement in angulated intrahepatic bile ducts and enable DGT without prior tract dilation. Because the catheter is not intended for tract dilation, it may offer an advantage in negotiating angulated bile ducts while preserving access for GW manipulation and contrast injection. In this single case, the catheter enabled direct advancement over a 0.018-inch GW and successful establishment of DGT, followed by plastic stent placement without additional dilation, thereby facilitating the completion of fine-gauge EUS-HGS. The target duct in this case was ∼5 mm in diameter and was already dilated due to cholangitis and intrahepatic bile duct stones; therefore, further cases are needed to determine whether this approach is useful in truly nondilated intrahepatic bile ducts. This case suggests that the novel DLC may facilitate fine-gauge EUS-HGS by enabling establishment of the DGT without prior tract dilation.

## Supplementary Material

goag097_Supplementary_Data
