# Supplementary material for: Endoscopic ultrasound-guided hepaticogastrostomy with a 22-G needle and novel double-lumen catheter after pancreaticoduodenectomy
Source: Gastroenterol Rep (Oxf). 2026 Sep 18;14:goag097. doi: 10.1093/gastro/goag097 (PMC13588573; doi:10.1093/gastro/goag097)
Supplement: Supplementary file 1 [file goag097_supplementary_data.zip › Supplementary/Supplement Video title.docx]

Video 1. Endoscopic ultrasound-guided hepaticogastrostomy using a 22-G needle and a novel double-lumen catheter.
