# Supplementary material for: Endoscopic ultrasound-guided hepaticogastrostomy with a 22-G needle and novel double-lumen catheter after pancreaticoduodenectomy
Source: Gastroenterol Rep (Oxf). 2026 Sep 18;14:goag097. doi: 10.1093/gastro/goag097 (PMC13588573; doi:10.1093/gastro/goag097)
Supplement: Supplementary file 1 [file goag097_supplementary_data.zip › Supplementary/Supplementary_Figures.docx]

**Supplementary Figures**

**Supplementary Figure S1**. The novel uneven double-lumen catheter (Uneven double-lumen catheter 18 type; PIOLAX Medical, Kanagawa, Japan) is compatible with a 0.018-inch guidewire and features a tapered tip to minimize the step-off between the guidewire and the catheter. A second guidewire can be inserted via an additional lumen located 5 mm proximal to the tip, allowing rapid transition to a double-guidewire technique without prior tract dilation.

Alt text: Photograph of the novel uneven double-lumen catheter featuring a tapered tip compatible with a 0.018-inch guidewire and an additional lumen for placement of a second guidewire.

**Supplementary Figure S2**. Preprocedural computed tomography images. Dilation of the intrahepatic bile ducts and intrahepatic bile duct calculi (Red arrow). (A) Axial image. (B) Coronal image.

Alt text: Computed tomography images of dilated intrahepatic and multiple intrahepatic bile duct stones indicated by red arrows.
